# The history and epidemiology of Middle East respiratory syndrome corona virus

**DOI:** 10.1186/s40248-017-0101-8

**Published:** 2017-08-07

**Authors:** Aisha M. Al-Osail, Marwan J. Al-Wazzah

**Affiliations:** Imam abdulrahman Alfaisal University (University of Dammam previously), Prince Saud bin Fahd Street, P.O. Box 3669, Khobar, 31952 Saudi Arabia

**Keywords:** Corona virus, Bat, Camel, Middle East respiratory syndrome corona virus, Saudi Arabia

## Abstract

Corona viruses cause common cold, and infections caused by corona viruses are generally self-resolving. During the last 4 years, corona viruses have become the most important viruses worldwide because of the occurrence of several recent deaths caused by corona viruses in Saudi Arabia. Spread of the infection occurred worldwide; however, most cases of mortality have occurred in the Middle East. Owing to the predominance of outbreaks in the Middle Eastern countries, the virus was renamed a Middle East respiratory syndrome corona virus (MERS-CoV) by the Corona virus Study Group. The Center for Diseases Control and Prevention and World Health Organization maintain a website that is updated frequently with new cases of MERS-CoV infection. In this review, we describe the history and epidemiology of this novel virus. Studies of the genetics and molecular mechanisms of this virus are expected to facilitate the development of vaccines in the future.

## Background

The first cases of corona virus infection in Saudi Arabia, specifically Jeddah, were reported on June 13, 2012; after this outbreak, corona virus continued to spread overseas to many countries in Asia, Africa, Europe, and America [1–4]. During this outbreak, most cases occurred in Middle Eastern countries, including those in the Gulf region (Saudi Arabia, Qatar, United Arab Emirates, Oman, Bahrain, Kuwait, and Iraq), as well as Jordan, Syria, Lebanon, Palestine, and Egypt. These countries were considered to be at high risk for corona virus infection according to the European Centre for Disease Prevention and Control (ECDC). Thus, any person arriving from any of these countries should be screened at the airport before entering, particularly after several cases of infection were reported in European countries, including France and the United Kingdom [4–6].

The corona virus responsible for this outbreak was a novel virus that mainly affected adults. The transmission mechanism and potential treatment strategies were still unclear. Notably, although this virus initially appeared to only affect adults, cases have also been observed in pediatric patients. Thus, this novel, potentially fatal virus represented a substantial public health risk. In this review, we discuss the history, epidemiology, and molecular mechanisms of this novel virus, called the Middle East respiratory syndrome corona virus (MERS-CoV).

## Study design

This study was scheduled and directed to be adhered to PRISMA standards of quality for reporting meta-analysis. We used PRISMA 2009 checklist in writing all parts of the article. It was a meta- analysis.

### Eligibility criteria

#### A. Inclusion criteria

1. Article discussed the corona virus history and epidemiology.
2. Case reports or studies involving patients who were exposed to corona virus.
3. All age group.
4. Article published within last 4 years.

#### B. Exclusion criteria

Suspected case with no proves.

## Study selection

The two authors independently screened all titles and abstracts, evaluated both the full-text of eligible publications and the risk of bias of included studies, and extracted data (Table 1).

**Table 1:** PHASE 3 Judging risk of bias

Domain	Concern	Rationale for concern
Concerns regarding specification of the study eligibility criteria	Non –confirmed cases	Decrease the risk of bias (false positive)
Concerns regarding methods used to identify and/or select studies.	None	None
Concerns regarding used to collect data and appraise the studies	Repeated article	Increase the false positive results
Concerns regarding the synthesis and findings	Different finding between the article	To direct the results toward the major finding and analyzed it
Describe whether conclusions were supported by the evidence:		
A. Did the interpretation of findings address all of the concerns identified in Domain 1 to 4?		Yes
B. Was the relevance of identified studies to the review’s research question appropriately considered?		Probably yes
C. Did the reviewers avoid emphasizing results on the basis of their statistical significance?		Yes
Risk of bias in the review		Risk: Low

### Data collection process

The authors searched the topic through the following steps

- Search in Google.
- Search in PubMed
- Evaluated the papers, divided them depending on the inclusion and exclusion criteria.
- Evaluated the articles and analyzed them.

### Data items

We searched through Google, Pub Med, Pub Med Central, CAS, Citebase, DOAJ, Embase, Embiology, MEDLINE, OAIster, SCImago, Scopus, SOCOLAR and Zetoc by using the keywords.

### Risk of bias in individual studies

We used the ROBIS tool and guidance which was available in the website (www.robis-tool.info) and (www.jclinepi.com). The risk of bias was low (Table 3).

## History of the corona virus

**Fig. 1:**
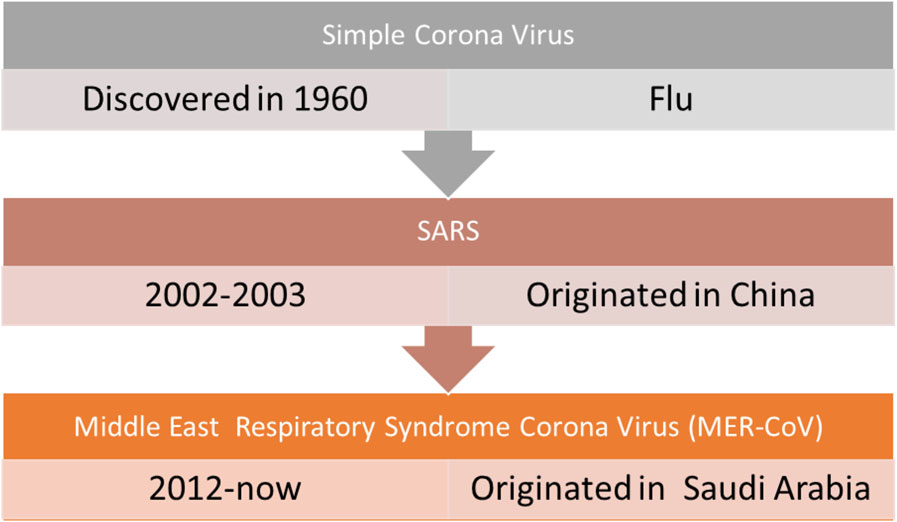
Summary of Corona Virus history

Corona virus was first identified as a cause of the common cold in 1960. In one study carried out in Canada in 2001, more than 500 patients presented with flu-like symptoms. Virological analyses showed that 3.6% of these cases were positive for the HCoV-NL63 strain by polymerase chain reaction (PCR). Until 2002, corona virus was considered a relatively simple, nonfatal virus; however, an outbreak in 2002–2003 in Guangdong province in China, which resulted in spread to many other countries, including Thailand, Vietnam, Taiwan, Hong Kong Singapore, and the United States of America, caused severe acute respiratory syndrome (SARS) and high mortality rates in over 1000 patients. After this outbreak, microbiologists and infectious disease experts focused on the understanding the pathogenesis of the disease and discovered that this infection was caused by a new form of corona virus. A total of 8096 individuals were infected with this virus, resulting in 774 deaths; thus, in 2004, the Centers for Disease Control and Prevention (CDC) and World Health Organization (WHO) declared a state of emergency [7–9]. In another report from Hong Kong, 50 patients presented with SARS, and more than 60% of these patients were positive for corona virus [10]. The evolution of this virus demonstrated that coronavirus is not a stable virus and can adapt to become more virulent, even lethal, to humans. Indeed, another outbreak in Saudi Arabia in 2012 resulted in many deaths and spread first to other countries in the Middle East and then worldwide, resulting in renewed interest in studies of this new form of coronavirus (Fig. 1) [7–10].

## Microbiology

**Fig. 2:**
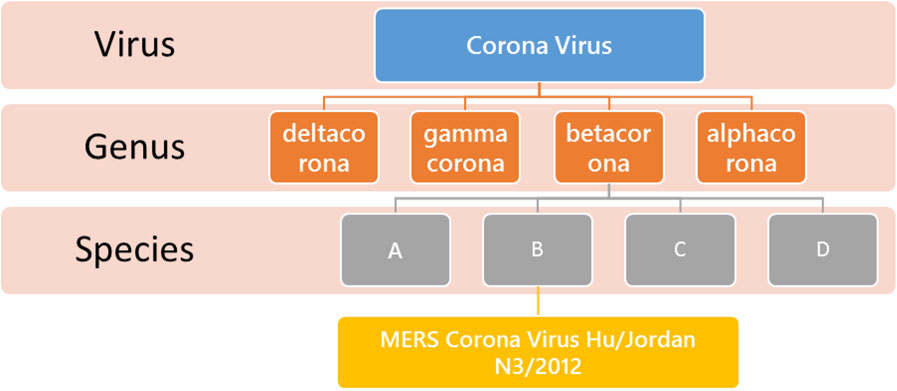
Simple corona virus microbiology

Corona virus is a single-stranded, enveloped RNA virus 1 that is spherical or pleomorphic in shape with bear’s club-shaped glycoprotein projections. There are subtypes of corona virus, alpha corona virus, beta corona virus, gamma corona virus, and delta corona virus, and each subtype has many serotypes. For example, OC43-like and 229E–like have been shown to affect humans, whereas the other types mainly affect animals (Fig. 2). Corona viruses are transmitted via airborne zoonotic droplets, and viral replication occurs in the ciliated epithelium, resulting in cellular damage and inflammatory reactions at the site of infection [3, 4]. In addition to humans, corona viruses are also found in bats, whales, pigs, birds, cats, dogs, and mice [11–15].

## Clinical presentation and investigational methods

The median incubation period for MERS-CoV is 5.2–12 days, and adults are mostly affected. However, several pediatric cases have been reported in Saudi Arabia. The clinical presentation of MERS-CoV ranges from flu-like symptoms, i.e., fever and cough in 87% of patients, chills, rigor, rhinorrhea, myalgia, and fatigue, to more severe symptoms, including shortness of breath in 48% of patients and respiratory failure, resulting in the requirement for intubation and ventilation. Gastrointestinal symptoms, including nausea, vomiting, diarrhea, and abdominal pain, have also been reported in about 35% of cases, and acute renal failure necessitating hemodialysis has been seen. On physical examination, patients generally present with fever and pulmonary findings, such as rhonchi and crepitation. Laboratory investigation may revealed lymphopenia, thrombocytopenia, disseminated intravascular coagulation (DIC), and multi-organ failure, which can result in death. The CDC recommends collecting multiple specimens at different times from different sites; for example, samples should be collected from oropharyngeal and nasopharyngeal swabs, sputum, blood, and stool and rectal swabs, as well as the lower respiratory tract, which is most frequently positive for the virus.

Typically, samples will be processed by real-time reverse transcription (rRT)-PCR to analyze the following sequences: upstream of the E protein gene (upE), open reading frame 1b (ORF 1b), and open reading frame (ORF 1a), which is the most sensitive sequence for identification of the specific virus. RNA dependent RNA polymerase (RdRp) and N genes are also target sites in the MERS-CoV genome. To confirm the diagnosis, one of the following criteria must be present: (1) a positive PCR result for at least two different specific targets in the MERS-CoV genome or (2) one positive PCR result for a specific target on the MERS-CoV genome and an additional different PCR product confirming a known sequence of MERS-CoV. The algorithm for diagnosis is as follows: (A) upE-specific rRT-PCR – if positive, confirm with ORF 1a rRT-PCR analysis, and, if positive, the case is confirmed; (B) upE-specific rRT-PCR – if positive, confirm with sequencing of one of the two target sites (RdRp or N assay), and, if positive, the case is confirmed. False negative results can occur due to issues with specimen collection, including early or late collection or shipping/handling problems [16–23].

## Corona virus in the Middle East and Saudi Arabia

In June 13, 2012, the first reported case of MERS-CoV 1 occurred in Jeddah, Saudi Arabia. This outbreak resulted in many cases of infection, particularly among health care workers who were in direct contact with the patients, indicating that the virus could be transmitted from human to human via air droplets. This prompted strict contact precautions, such as isolation and the use of personal protective equipment, i.e., gloves, N 95-typemasks, and gowns. In Saudi Arabia, many cases of MERS-CoV were reported in almost all provinces, with Jeddah, Makka, Riyadh, and Al-Hassa being the most commonly affected cities. Additionally, many outbreaks were reported in hospitals, causing affected hospitals to close down and not to accept any infected patients. Most of the patients who died were immunocompromised, having conditions such as chronic renal failure, congestive heart failure, and diabetes or having recently received organ transplants (e.g., kidney transplants). After the initial outbreak in Saudi Arabia, MERS-CoV was reported in several other countries, including Qatar, Bahrain, Kuwait, Jordan, and Tunisia. According to the Minister of Health in Saudi Arabia, from June 13, 2012 until December 2015, a total of 1227 cases of MERS-CoV have been reported, with 728 recovered, one still under treatment, and 549 expired due to MERS-CoV-related symptoms. The Minister of Health, primary healthcare facilities, and public health officials immediately sought to educate the population in Saudi Arabia through internet, lectures, and brochures and continued to carefully record each new case in order to improve knowledge and therapeutic strategies for this virus. The latest report from the WHO on December 7, 2015 showed that MERS-COV has been identified in 26 countries, with 1621 confirmed cases and 584 deaths globally. Notably, the highest numbers of both reported cases and deaths have been in the Saudi Arabia [1, 2, 24, 25].

## MERS-CoV and bats

Corona virus as known to be a zoonotic virus; however, the MERS-CoV is a novel virus, and whether zoonotic transmission occurs is not clear yet. International studies carried out from 2012 to 2014 in Mexico, European countries (i.e., Germany, Ukraine, the Netherlands, and Romania), Ghana, and South Africa have examined whether bats may be carriers of MERS-CoV. These studies have tested bats mainly for the 329-bp fragment of RdRp using blood, fecal, and oral samples (Table 2). The bat species that were tested in these studies included *Pipistrelluspipistrellus*, *P. nathusii*, *P. pygmaeus*, Nycteris, and Neoromiciazuluensis, and 5.3–24.9% were found to be positive for MERS-CoV, with most positive results (> 70%) being identified in fecal samples with high viral loads [26–29]. Thus, it may be possible for transmission to occur via bats; however, in Saudi Arabia, the species of bats that patients may have come in contact with are different from those tested, including Rhinopomahardwickii, Rhinopomamicrophyllum, Taphozousperforatus, *P. kuhlii*, Eptesicusbottae, *Eidolon helvum*, and Rosettusaegyptiacus. Thus, although there was a positive association between bats and corona virus infection, there was no association between bats and MERS-CoV. Therefore, these data have suggested that MERS-CoV is not transmitted through bats [30–33].

**Table 2:** Summary of international and national studies conducted to date to identify the link between bats and MERS-CoV. All studies were conducted between 2012 and 2014, and positive results were obtained from rectal swabs

Study location	Sample number	Species type	Gene detected	Types of corona	Family of bat species
Mexico	606	42	329-bp fragment of the RdRp	4.45%	*Phyllostomidae*
					*Mormoopidae*
					*Molossidae*
					*Vespertilionidae*
					*Emballonuridae*
Ghana and Europe	5030	10	EMC/2012	14.7%–24.9%	*P.pipistrellus*,
					*P. nathusii*,
					*P. pygmaeus*
					*Nycteris*
South Africa	62	13	329-bp fragment of the RdRp	8%	*Neoromicia* cf. *zuluensis*
Saudi Arabia	96	7	329-bp fragment of the RdRp	3.5%	*Rhinopomahardwickii*,
					*Rhinopomamicrophyllum*,
					*Taphozousperforatus*,
					*Pipistrelluskuhlii*,
					*Eptesicusbottae*,
					*Eidolon helvum*,
					*Rosettusaegyptiacus*

## MERS-CoV and camels

Researchers have also examined whether camels may be linked to the outbreak of MERS-CoV in Saudi Arabia. Studies have been carried out in many Middle Eastern countries, including Saudi Arabia, Qatar, Egypt, United Arab Emirates, and Oman, using samples from lung, nasal, and rectal swabs. Positivity for MERS-CoV by RT-PCR for the RdRpwas observed in 1.6–61.5% of samples, mostly lung and nasal swabs (Table 3). Analyses using anti-MERS-CoV antibodies have shown that 98–100% of camels are positive for MERS-CoV; consistent with this, the incidence of MERS-CoV in humans is 15 times higher in camel shepherds and 23 times higher in slaughterhouse workers than in the general population. Therefore, these data supported that the main route of transmission from camels to humans is through the respiratory system [34–43].

**Table 3:** Summary of studies investigating the link 1 between camels and MERS-CoV in Middle East. All studies were conducted between 2013 and 2014

Study location	Sample number	Positive by RT-PCR	Species	Sample sites
Egypt and Hong Kong	110	Not mentioned^a^	Not mentioned	Serum
Jordan	11	Not mentioned^a^	Not mentioned	Serum and rectal
Oman				
Qatar	33	0–58%	Not mentioned	Serum, nasal swabs, rectal swabs, and milk
United Arab of Emirates	651	59.8% in serum	*Camelusdromedarius*	Fecal samples and serum
		1.53% in fecal samples		
Saudi Arabia Al-Ahsa	96	29.2% in nasal swabs	*Camelusdromedarius*	Nasal and lung swabs
		61.5% in lung tissue		

^a^The main aim of this study was to report MERS-CoV antibodies; however, the virus itself was not tested (range: 98–100%)

## Management and vaccination

The main treatment strategy for typical corona virus infection is supportive therapy, in deeding administration of antipyretics and analgesics, maintenance of hydration, respiratory support by either mechanical ventilation or extracorporeal membrane oxygenation (ECMO), and treatment with antibiotics in the case of bacterial super infections. However, such treatments may not be sufficient for MERS-CoV infections, which may be more severe. Ribavirin and interferon alpha have been shown to have synergistic effects and are more beneficial when started early. Additionally, mycophenolic acid has been shown to be efficacious and can be used as a monotherapy; however, initial clinical trials included few patients, and further studies are necessary [44–49]. Although several companies are attempting to develop MERS-CoV vaccines, none are available yet. Improving our understanding of viral antibodies will facilitate the design of appropriate and efficacious vaccines.

## Conclusion

MERS-CoV is a lethal zoonotic virus that originated in the Middle East. The main source of transmission, as has been shown in several studies, is through camels. Therapies are still under development and include ribavirin a, interferon alpha, and mycophenolic acid. Further studies are underway to develop an effective vaccine for MERS-CoV aiming to reduce the incidence and mortality rate of infection with this virus.

## Abbreviations

CDC: Centers for Disease Control and Prevention
DIC: Disseminated 1 Intravascular Coagulation
ECDC: European Centre for Disease Prevention and Control
ECMO: Extra Corporeal Membrane Oxygenation
MERS-CoV: Middle East respiratory syndrome corona virus
ORF: Open Reading Frame
PCR: Polymerase Chain Reaction
RdRp: RNA-dependent RNA polymerase
rRt: real-time reverse transcription.
SARS: Severe Acute Respiratory Syndrome (SARS)
WHO: World Health Organization
